# Investigating the Potential Anti-Alzheimer’s Disease Mechanism of Marine Polyphenols: Insights from Network Pharmacology and Molecular Docking

**DOI:** 10.3390/md21110580

**Published:** 2023-11-06

**Authors:** Kumju Youn, Chi-Tang Ho, Mira Jun

**Affiliations:** 1 Department of Food Science and Nutrition, Dong-A University, Busan 49315, Republic of Korea; kjyoun@dau.ac.kr; 2 Department of Food Science, Rutgers University, New Brunswick, NJ 08901, USA; ctho@sebs.rutgers.edu; 3 Department of Health Sciences, The Graduate School of Dong-A University, Busan 49315, Republic of Korea; 4 Center for Food & Bio Innovation, Dong-A University, Busan 49315, Republic of Korea

**Keywords:** Alzheimer’s disease, marine polyphenols, eckol, dieckol, 8,8′-bieckol, network pharmacology, docking simulation

## Abstract

Marine polyphenols, including eckol(EK), dieckol(DK), and 8,8’-bieckol(BK), have attracted attention as bioactive ingredients for preventing Alzheimer’s disease (AD). Since AD is a multifactorial disorder, the present study aims to provide an unbiased elucidation of unexplored targets of AD mechanisms and a systematic prediction of effective preventive combinations of marine polyphenols. Based on the omics data between each compound and AD, a protein–protein interaction (PPI) network was constructed to predict potential hub genes. Gene Ontology (GO) and Kyoto Encyclopedia of Genes and Genomes (KEGG) pathway analyses were performed to provide further biological insights. In the PPI network of the top 10 hub genes, AKT1, SRC, EGFR, and ESR1 were common targets of EK and BK, whereas PTGS2 was a common target of DK and BK. GO and KEGG pathway analysis revealed that the overlapped genes between each compound and AD were mainly enriched in EGFR tyrosine kinase inhibitor resistance, the MAPK pathway, and the Rap1 and Ras pathways. Finally, docking validation showed stable binding between marine polyphenols and their top hub gene via the lowest binding energy and multiple interactions. The results expanded potential mechanisms and novel targets for AD, and also provided a system-level insight into the molecular targets of marine polyphenols against AD.

## 1. Introduction

Alzheimer’s disease (AD) is a neurodegenerative disease of the central nervous system and the most prevalent cause of dementia in the elderly. Approximately 50 million people worldwide live with dementia, and owing to the aging population, the number of patients is expected to triple by 2050, increasing the risk of disability, disease burden, and healthcare costs [1]. Typical pathological features of AD are senile plaque accumulation resulting from extracellular deposition of amyloid-beta (A*β*), and neurofibrillary tangles composed mainly of hyperphosphorylated tau protein. Moreover, the disruption of neuronal synapses, accumulation of oxidative stress, mitochondrial dysfunction, and neuroinflammation are associated with AD occurrence and development [2]. Because AD has a long prodromal phase and current treatment strategies improve symptoms without altering the underlying course of the disease, early prevention appears to be particularly important in slowing disease progression.

Marine algae have attracted considerable attention as natural sources of bioactive ingredients that play an important role in the development of functional foods [3]. In addition, they grow in oceans, which cover approximately 70% of the Earth’s surface, and have relatively low environmental impacts compared to other food sources. As the global population increases and more resources are needed for climate resilience, marine algae could become a sustainable food source for the future [4]. They can be divided into three classes based on their pigmentation: brown (Phaeophyceae), red (Rhodophyceae), and green (Chlorophyceae). Brown is reported to be the richest in terms of structural diversity, including terpenoids, polyphenols, polysaccharides, sterols, and peptides, which have various biologically active properties [5].

Marine polyphenols are unique metabolites of brown algae species not found in terrestrial plants. In particular, phlorotannins are an important class of marine polyphenols, constituting approximately 5–12% of their dry weight [6]. They are formed by the polymerization of phloroglucinol (1,3,5-trihydroxybenzene) units, among which eckol(EK), dieckol(DK), and 8,8′-bieckol(BK) have been found to be the major compounds [7]. These compounds are considered to be functional food ingredients with a wide range of biological activities, including antioxidant, anti-inflammatory, antihypertensive, antidiabetic, and antitumor neuroprotective effects, among others [8]. Recently, the neuroprotective effects of these major phlorotannins have attracted considerable attention [9,10,11,12,13,14]. Three compounds exerted potent inhibitory effects on cholinesterases, monoaminoxidase, and *β*-secretase (BACE1), which are known as AD-related enzymatic targets [9,10,11,12]. They inhibited proinflammatory cytokines (TNF-*α*, IL-*β*1) and mediator (PGE2) through the NF-*κ*B/MAPK signaling pathway in the PC12 cell line [13]. A recent study showed that DK protected both murine hippocampal HTT22 and primary cortical neurons against glutamate-induced neurotoxicity by reducing ROS levels and activating the nuclear factor-like 2/heme oxygenase-1 (Nrf-2/HO-1) pathway [14]. Marine polyphenols have been investigated for various biological properties, but a study of their neuroprotective effects is needed to demonstrate their potential in the prevention of AD.

Network pharmacology is an emerging and promising integrated strategy that is based on systems biology, bioinformatics, and multivariate pharmacology. This strategy is a novel approach to analyzing candidate mechanisms for multifactorial diseases, identifying new targets, and expanding new indications at a systematic level, which can comprehensively reflect the mechanism of potential candidates on disease networks, thus supporting lead compound discovery for complex diseases and natural products [15].

**Figure 1:**
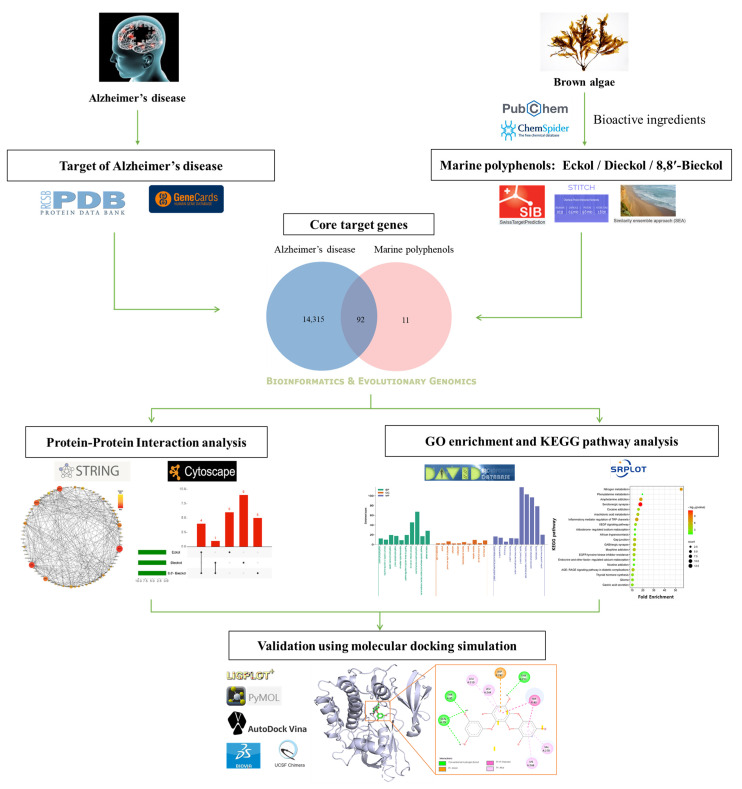
Systematic strategy workflow for the prediction of the preventive mechanisms of marine polyphenols in Alzheimer’s disease.

The present study demonstrated the novel implications on EK, DK, and BK of important genes and pathways that could potentially be exploited against AD. Based on the intersection targets between each marine polyphenol and AD, a protein–protein interaction (PPI) network was constructed to discover hub genes and subnetworks. For discovering the various mechanisms of the three compounds for AD prevention, Gene Ontology (GO) and the Kyoto Encyclopedia of Genes and Genomes (KEGG) pathways were subsequently conducted. Finally, the relationship between key targets and each compound was validated by molecular docking simulation. Overall, the results were first to explore the novel target genes, and their AD-related mechanisms, of marine polyphenols via network pharmacology. The workflow involved in the present study is illustrated in Figure 1.

## 2. Results

### 2.1. Core Gene Targets of Marine Polyphenols and AD

**Figure 2:**
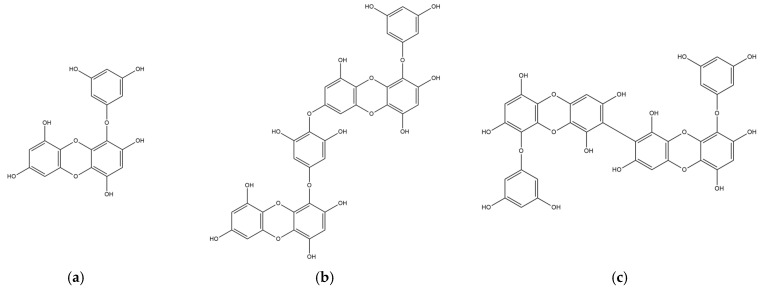
2D chemical structures of marine polyphenols including (**a**) EK, (**b**) DK, and (**c**) BK.

**Figure 3:**
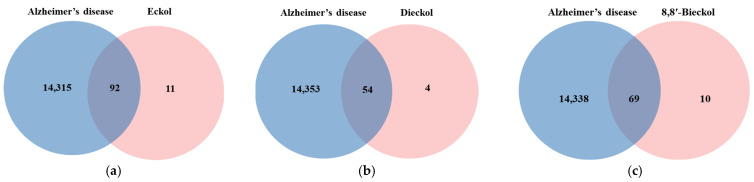
Venn diagram for the interaction between Alzheimer’s disease and marine polyphenols (**a**) EK, (**b**) DK, and (**c**) BK.

The chemical structures of marine polyphenols, EK, DK, and BK are presented in Figure 2. A total of 103, 58, and 79 potential targets were identified for EK, DK, and BK, respectively, using STITCH and Swiss Target prediction web tools (Supplementary Table S1). The GeneCards database provided a total of 14,407 AD-related gene targets demonstrating the complexity of this disease (Supplementary Table S2). Additionally, the overlapped genes between AD and each compound were 92 for EK, 54 for DK, and 69 for BK in Venn diagram (Figure 3), suggesting that these genes were proven to be the core targets of marine polyphenols for AD prevention and should be considered for subsequent PPI analysis.

### 2.2. PPI Networks of Marine Polyphenols

**Figure 4:**
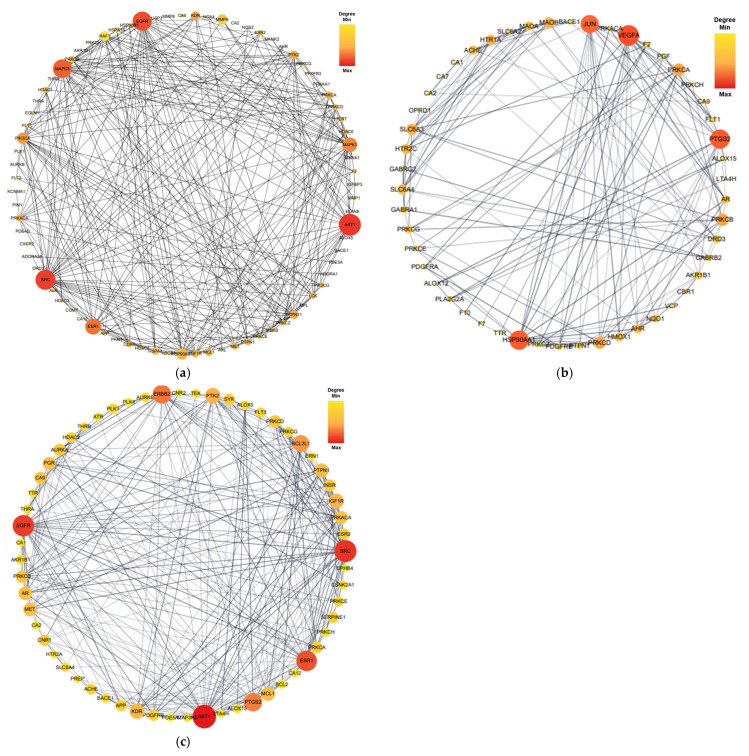
The protein–protein interaction network of marine polyphenols of anti-Alzheimer’s disease targets. (**a**) EK-AD, (**b**) DK-AD, and (**c**) BK-AD. The circles (nodes) reflect the anti-AD targets, the color (yellow-orange-red) and size of the circle is proportional to its intensity. The line (edges) represent the target protein interactions.

Proteins form dynamic PPI networks to attain multifunctionality and various cellular signaling pathways. To obtain the PPI networks of EK, DK, and BK with AD, the core targets of 92 (EK), 54 (DK), and 69 (BK) were imported into the STRING database and visualized using Cytoscape software (version 3.10.0). As shown in Figure 4, nodes (circles) in the PPI network represent target molecules (e.g., genes or proteins), and edges (connecting lines) represent various molecular relationships to support the understanding of the functional organization of the proteome and cellular process in AD progression [16]. The PPI network of EK-AD consisted of 79 nodes and 564 edges with an average degree of 14.28. In this network of DK-AD, 50 nodes and 166 edges with an average degree of 6.64 were included. In addition, the PPI network BK-AD contained 60 nodes and 295 edges with an average degree of 9.83. Among the nodes of each compound, the top 10 targets with a higher degree of connectivity were selected as hub genes and shown in Table 1. Interestingly, for the top three hub genes, EK and BK exactly shared AKT1, SRC, and EGFR genes for AD prevention. In contrast, DK occupied somewhat different ones, such as VEGFA, PTGS2, and HSP90AA1, in the AD prevention network.

**Figure 5:**
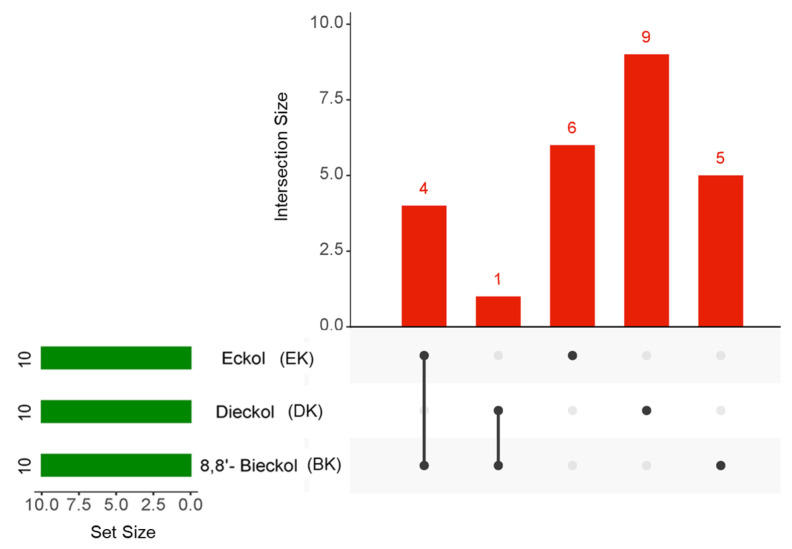
The UpsetR plot for the intersection of the top 10 hub genes in marine polyphenols. The green horizontal bars and the red vertical bars represent the number of each target and the number of the intersection targets of the three marine polyphenols, respectively.

To obtain an intersection of the top 10 hub genes of the three compounds in AD, an UpsetR diagram was constructed (Figure 5). The common targets of EK and BK in AD were demonstrated to be AKT1, SRC, EGFR, and ESR1. PTGS2 was considered a common target of DK and BK (Table 2). These results suggested that EK and BK might have similar mechanisms of action, while DK might exert somewhat different ones on AD prevention.

### 2.3. GO Enrichment and KEGG Pathway Analysis of Marine Polyphenols

**Figure 6:**
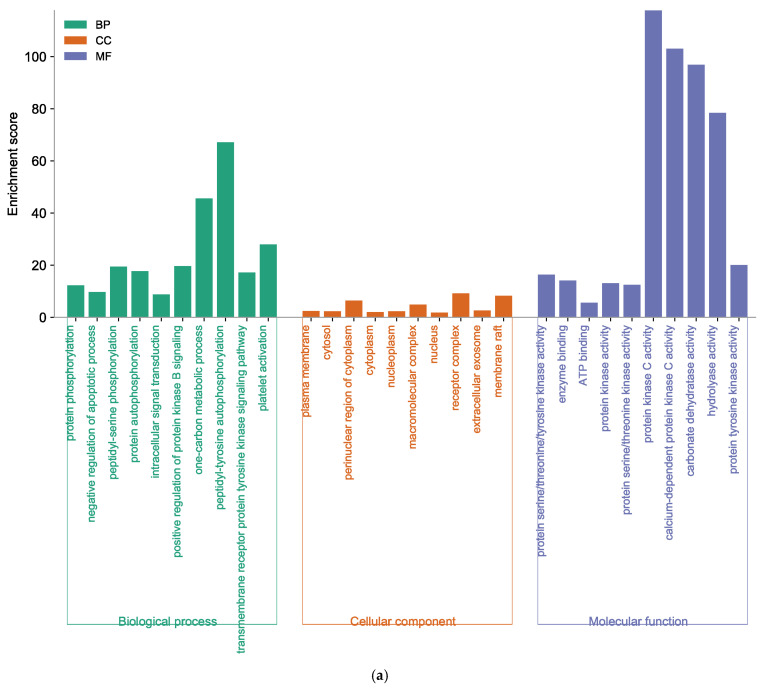

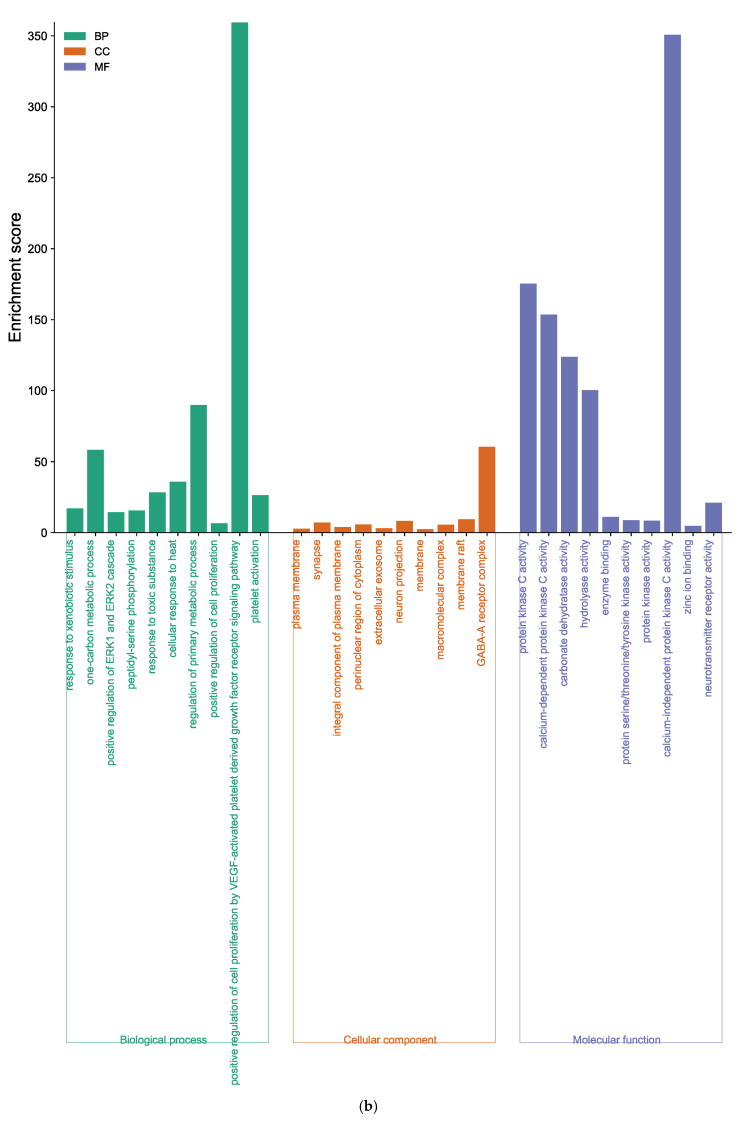

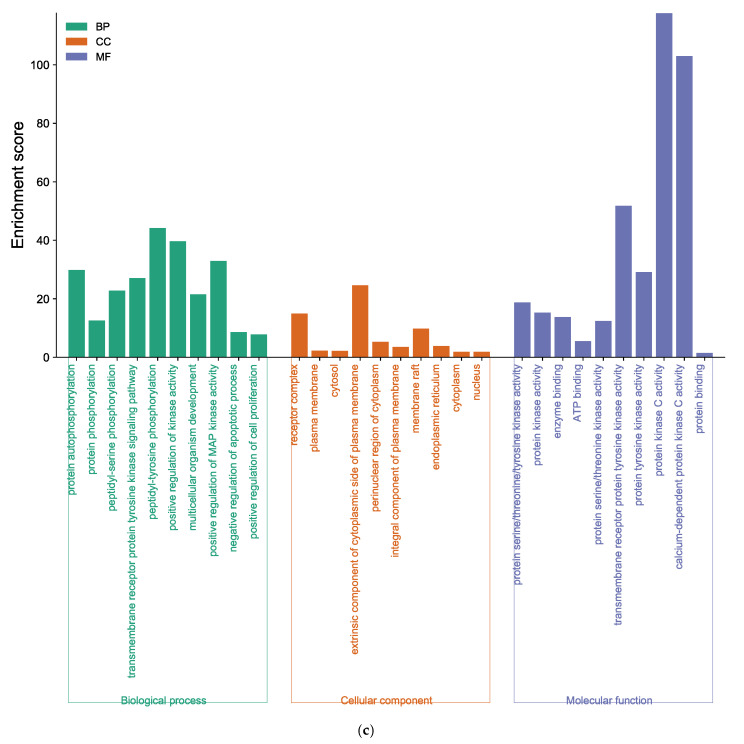
Function of targets with gene ontology (GO) enrichment analysis of (**a**) EK, (**b**) DK, and (**c**) BK. The biological process (BP), cell composition (CC), and molecular function (MF) of the GO enrichment analysis are represented by green, orange, and blue columns, respectively.

For further elucidation of the biological functions and potential signaling pathways of marine polyphenols for AD prevention, GO and KEGG analyses were conducted. The GO enrichment histograms showed the top 10 enriched categories in biological process (BP), cellular component (CC), and molecular function (MF), in ascending order of *p*-values (Figure 6). The core targets of EK were involved in 365 BP terms, 73 CC terms, and 107 MF terms (Supplementary Tables S3–S5). In the BP analysis, the key target genes of EK were mainly enriched in protein phosphorylation, negative regulation of the apoptotic process, peptidyl-serine phosphorylation, etc. (Figure 6a). The CC ontology identified significant enrichment for the plasma membrane, cytosol, perinuclear region of cytoplasm, etc. In addition, protein serine/threonine/tyrosine kinase activity, enzyme binding, ATP binding, protein kinase activity, etc., were mainly enriched for EK in the MF category.

GO analysis of DK-anti-AD showed that 155 BP terms, 42 CC terms, and 59 MF terms were enriched (Supplementary Tables S6–S8). The core targets of DK were involved in response to xenobiotic stimulus, the one-carbon metabolic process, positive regulation of ERK1 and ERK2 cascade, etc., in the BP category (Figure 6b). In the CC enrichment analysis, the core targets of DK were mainly found in the plasma membrane, synapse, integral component of the plasma membrane, and elsewhere. The MF analysis of DK suggested protein kinase C activity, calcium-dependent protein kinase C activity, carbonate dehydratase activity, and others.

The core target of BK-anti-AD was found to be associated with 268 BP terms, 62 CC terms, and 71 MF terms (Supplementary Tables S9–S11). As shown in Figure 6c, BP analysis results of BK, such as protein auto-phosphorylation and peptidyl-serine phosphorylation, were included. The enriched CC ontologies of BK were dominated by the receptor complex, plasma membrane, cytosol, etc. In addition, factors such as protein kinase activity, and the binding of enzyme and ATP were included in the MF enrichment of BK.

**Figure 7:**
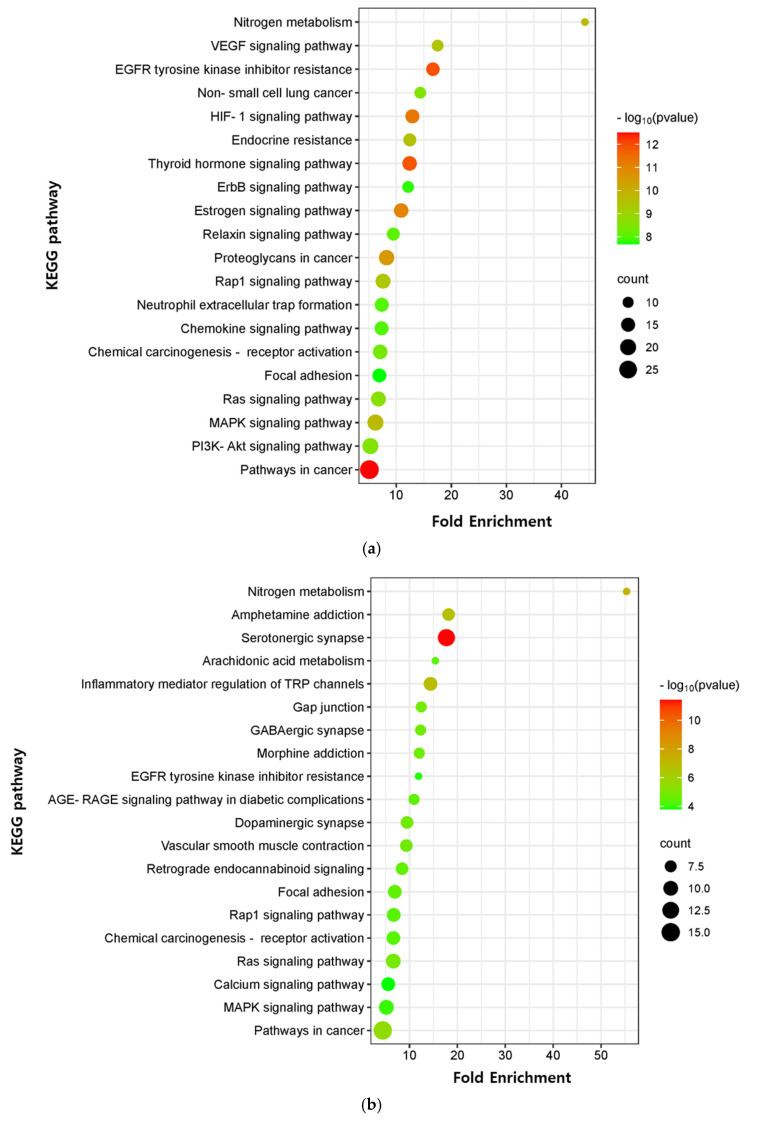

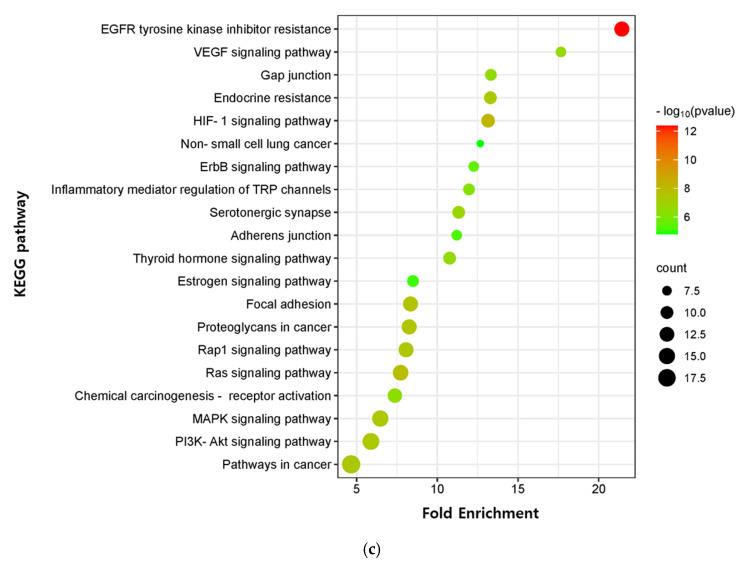
KEGG pathway enrichment analysis of (**a**) EK, (**b**) DK, and (**c**) BK. The *x*-axis represents the gene ratio; the *y*-axis represents the enrichment pathway; the size of the dot represents the number of genes; the color of the dot represents the level of *p*-value.

The critical signaling pathway associated with our three compounds in AD was analyzed using the KEGG pathway (Supplementary Table S12). The top 20 pathways were chosen to construct bubble maps with a threshold of *p* < 0.05 (Figure 7). In total, 150, 75, and 115 significant pathways were exported for EK, DK, and BK, respectively. EK mainly acted on pathways in cancer, EGFR tyrosine kinase inhibitor resistance, and the thyroid hormone signaling pathway, while DK principally affected the serotonergic synapse, nitrogen metabolism, and the inflammatory mediator regulation of TRP channels. KEGG pathways for BK were principally enriched in EGFR tyrosine kinase inhibitor resistance, the HIF-1 signaling pathway, and the Ras signaling pathway. In addition, pathways in cancer, EGFR tyrosine kinase inhibitor resistance, MAPK signaling pathway, Rap1 and Ras signaling pathway, chemical carcinogenesis, and focal adhesion were common pathways for these three compounds (Table 3). It was found that the hub genes of marine polyphenols were closely related to these top 20 pathways, demonstrating these hub genes of our compounds might play a role in AD prevention.

### 2.4. Molecular Docking Validation of Marine Polyphenols

**Figure 8:**
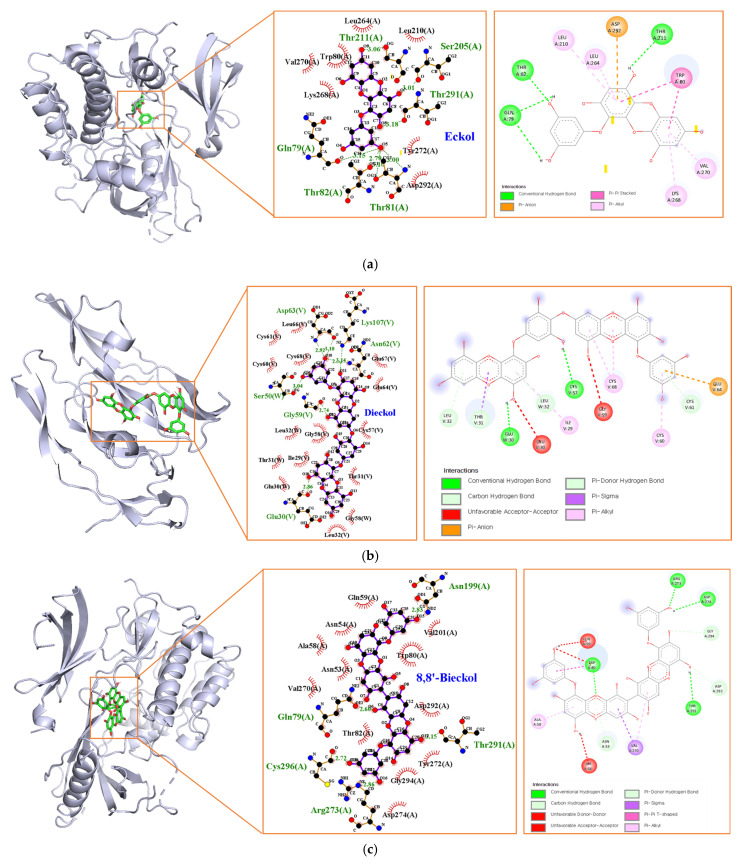
Molecular docking analysis of (**a**) EK, (**b**) DK, and (**c**) BK.

Marine polyphenols were docked with their top-ranked hub genes (AKT1 and VEGFA) to validate the network pharmacology results. The results of the molecular docking and binding energies are presented in Figure 8 and Table 4. EK, DK, and BK had a strong binding affinity of −9.7, −10.1, and −11.3 kcal/mol, respectively. The EK-AKT1 complex formed hydrogen bonds with residues Gln79, Thr81, Thr82, Ser205, Thr211, and Thr291; Pi interacted with residues Trp80, Leu210, Leu264, Lys268, Val270, and Asp292; and van der Waals forces with residues Trp80, Leu210, Leu264, Lys268, Val270, Tyr272, and Asp292. The binding affinity of the DK-VEGFA complex was attributed to hydrogen bonds with residues Glu30, Ser50, Gly59, Asn62, Asp63, and Lys107; pi interactions with residues Ile29, Thr31, Leu32, Cys60, Cys61, Glu64, and Cys68; and van der Waals forces with residues Ile29, Glu30, Thr31, Leu32, Cys57, Gly58, Cys60, Cys61, Glu64, Leu66, Glu67, and Cys68. BK interacted with AKT1 by forming hydrogen bonding with Gln79, Asn199, Arg273, Thr291, and Cys296; pi interactions with Asn53, Ala58, Val270, Asp292, and Asp294; van der Waals forces with Asn53, Asn54, Ala58, Gln59, Trp80, Thr82, Val201, Val270, Tyr272, Asp274, Asp292, and Gly294. The Root Mean Square Deviation (RMSD) measurement is the most fundamental indicator used to assess stability of a protein–ligand complex. The results of redocking showed that the RMSD value of the three compounds were less than 2 Å, demonstrating the docking system of our compounds was acceptable and stable [17].

## 3. Discussion

AD is a complex multifactorial neurodegenerative disorder and the most common form of dementia. Despite all scientific efforts and many protracted and expensive clinical trials, no new drug has been approved by the FDA for treatment of AD since 2003. Indeed, more than 200 investigational programs have failed or have been abandoned in the last decade [18]. Therefore, it is crucial to investigate new approaches to the development of disease-modifying treatment for AD. So far, the strategies for AD therapy mainly focus on extracellular amyloid plaques and intracellular tau neurofibrillary tangles, the primary histopathological lesions of AD. In silico network pharmacology is emerging as a possible alternative to bridge the gap between experiments and clinical trials, thus reducing the overall cost, time and number of experiments. The present study aims to explore the novel target genes, and their AD-related mechanisms, of marine polyphenols via network pharmacology and validate by molecular docking analysis via an in silico system, providing theoretical support and directions for further in vitro and in vivo experimental research.

Marine polyphenols have attracted a lot of attention, primarily due to their polypharmacologic mechanisms of action. However, there have been challenges in attaining complete knowledge of their multi-targets, which is lacking compared to terrestrial polyphenols. The present study might suggest a new perspective on the mechanisms of action in AD prevention compared with previous experimental studies.

Phlorotannins are marine polyphenols formed by the polymerization of phloroglucinol monomer units, which are characterized by three hydroxyl groups bound to a benzene ring skeleton. Even though phlorotannin content can vary depending on the species, region, and extraction technique, HPLC analysis has shown that EK, DK, and BK are the major compounds in edible brown algae, accounting for more than half of the total phlorotannins [7]. Structurally, EK consists of three phloroglucinols, whereas DK and BK are six-ringed phloroglucinol derivatives, which are two EK joined either asymmetrically or symmetrically, respectively. Previous studies have shown that their biological activity is related to the presence of additional hydroxyl groups and the degree of polymerization [19].

Based on the node degree, the average shortest path length, closeness centrality, and clustering coefficient in the PPI network and modules, the hub genes were ranked. It is noteworthy that major hub genes of EK and BK such as AKT1, SRC, EGFR, and ESR1 are mainly involved in AD prevention. Furthermore, the docking results demonstrated a strong binding affinity between AKT1, the identified core target, and both EK and BK. Several recent experimental studies have demonstrated the potential of AKT1 as a novel AD target that differs from traditional core targets related to A*β* and tau [20,21,22,23,24,25,26]. In AD, AKT1 plays a role as an upstream regulator in amyloidogenesis, fibrillary A*β*, the autophagy system, and oxidative stress [20,21,22,23]. AKT1 also plays an important role in modulating NF-*κ*B-dependent inflammatory gene transcription [24]. In our previous studies, EK and BK exerted a predominant inhibitory effect on activation of the enzyme for A*β* production, A*β*-mediated oxidative stress, and NF-*κ*B signaling pathway activation [11,13]. Although further experimental investigation is required to clarify that AKT1 is a novel target of two compounds for AD prevention, it appears that AKT1 plays an important role in the neuroprotective effects of both compounds.

AKT encoded by AKT1 is a serine/threonine kinase participating in the phosphoinositide 3-kinase (PI3K) signaling pathway, which is well-known to regulate various cellular functions such as growth, proliferation, migration, and differentiation [20]. Furthermore, previous studies have shown that the AKT/PI3K signaling pathway is closely related to the other key hub genes, SR999C, EGFR, and ESR1. This pathway is activated by EGFR and ESR1 in AD progress [25,26]. The SRC protein-tyrosine kinase family has been reported to participate in pathways such as survival, proliferation, and the regulation of gene expression via AKT, but the crosstalk between SRC and AKT in AD has not been explored as yet [27]. In the present study, PTGS2, also known as cyclooxygenase-2 (COX-2), was found to be the only target gene for DK and BK. Interestingly, it was experimentally validated that PTGS was a target of DK and BK in our previous in vitro study and other previous literature, supporting that the target prediction of marine polyphenols in the present study was reasonable and reliable [13,28,29,30]. Furthermore, the results of the common target genes suggested that synergistic effects might be expected when taking marine polyphenols as food or an extract.

DK showed somewhat different but very interesting results. The compound regulated specific target genes including vascular endothelial growth factor A (VEGFA) and heat-shock protein 90 alpha family class A member 1 (HSP90AA1), which are key regulators involved in angiogenesis. Regarding the study of VEGFA in AD, higher-baseline CSF VEGFA levels are associated with slower rates of hippocampal atrophy and slower rates of cognitive decline, particularly among individuals with elevated levels of AD biomarkers [31]. Moreover, VEGFA affects blood-brain barrier (BBB) permeability through its receptors. The association of DK with VEGFA in AD has not been studied to date, but it is possible that VEGFA is involved, based on previous findings that the compound crosses the BBB despite its relatively high molecular weight [32]. Wei et al. reported that Hsp90 encoded by HSP90AA is involved in the degradation of A*β* and tau through the proteasome system [33]. Other studies have shown that HSP90AA1 might be a key gene in the progression of inflammation, and Hsp90 inhibitors were considered to be potent inhibitors of the inflammatory response [34,35].

Previous studies have demonstrated that a compound with a binding energy less than −7.0 kcal/mol has a high binding affinity for the target protein [36]. The present docking results suggested that the DK-VEGFA complex had the lowest binding energy (−10.1 kcal/mol). There are multiple hydrogen bonding interactions, pi interactions, and van der Waals forces, resulting in the formation of tight and stable protein–ligand complexes after docking.

GO results indicated that the core genes of the three compounds were related to peptidyl-serine phosphorylation, which is one of the most predominant post-translational modifications that are fundamental in mediating various cellular functions [37]. Furthermore, KEGG enrichment analysis demonstrated that EK, DK, and BK regulated common mechanisms in AD prevention, including EGFR tyrosine kinase inhibitor resistance, the MAPK signaling pathway, the Ras family (Rap1 and Ras) signaling pathway, and focal adhesion. However, the genes involved in modulating these common mechanisms were found to be AKT1, SRC, EGFR, and ESR1 for EK and BK, while it was VEGFA, PTGS2, HSP90AA1, and JUN for DK. Given the critical role of the MAPK signaling pathway in modulating A*β* deposition, tau hyperphosphorylation, neuroinflammation, etc., the importance of MAPKs in AD pathogenesis is being increasingly recognized [38,39]. According to a previous in vivo study, the Ras family signaling cascade and focal adhesion were proven to control synaptic plasticity in AD, suggesting that our compounds may improve cognitive function [40,41].

Taken together, the present study, based on network pharmacology and docking analysis, systematically demonstrated the potential mechanisms of marine polyphenols against AD. Furthermore, the key target genes and signaling pathways of our compounds in AD prevention have never been reported in previous studies, suggesting a novel framework for further investigation of the anti-AD effects of marine polyphenols.

## 4. Materials and Methods

### 4.1. Target Identification of Marine Polyphenols

The SMILE features of EK, DK, and BK were obtained from the PubChem database. These features were then imported into Swiss Target Prediction (http://www.swisstargetprediction.ch, accessed on 13 June 2023) and STITCH (http://stitch.embl.de/, accessed on 13 June 2023) database to predict compound-related target genes. Only the genes from “Homo sapiens” were selected for the following analysis.

### 4.2. Acquisition of Core Target of Marine Polyphenols in AD

The AD-related genes were retrieved from GeneCards (https://www.genecards.org/, accessed on 13 June 2023) database, with the search term “Alzheimer’s disease.” GeneCards is a human gene database that performs gene-centric data integration from approximately 150 web sources, including genomic, transcriptomic, proteomic, genetic, clinical, and functional information. The final AD target genes were obtained after removing the repetitive elements. Then, the targets of each marine polyphenol and AD were overlapped using Venn diagram (https://bioinformatics.psb.ugent.be/webtools/Venn/, accessed on 13 June 2023). 

### 4.3. PPI Network Construction

Using STRING11.0 database (https://string-db.org/, accessed on 7 July 2023), the PPI interaction was constructed to investigate the interaction relationship between the targets that contribute to AD prevention among EK, DK, and BK. “Homo sapiens” was set as the species and the confidence level (≥0.04) was used as the criterion for evaluating the interaction score. In addition, the network topology parameters were analyzed via Network Analyzer in CytoScape3.10.0 software. The key topological parameters such as degree, average shortest path length, closeness centrality, and clustering coefficient were used to rank the core targets of the PPI network. The intersections of the top 10 hub genes of marine polyphenols were visualized by UpSet R package tool (http://www.bioinformatics.com.cn, accessed on 7 July 2023).

### 4.4. GO and KEGG Enrichment Analysis

GO and KEGG pathway enrichment analyses were performed using the Database for Annotation, Visualization, and Integrated Discovery (DAVID) database (https://david.ncifcrf.gov, accessed on 10 July 2023). The results of GO analysis were described in the BP, CC, and MF categories based on *p* < 0.05. Moreover, the results were visualized in a histogram form using the SRplot (http://www.bioinformatics.com.cn, accessed on 10 July 2023). In the KEGG study, the top 20 pathways were finally selected and the results were output in the form of bubble plots.

### 4.5. Molecular Docking Analysis

To confirm the accuracy of the network pharmacology prediction, the active compounds were used as ligands to perform molecular docking with the key target proteins which were identified by network pharmacology. The 3D structures of the ligands were obtained in SDF format from the PubChem (https://PubChem.ncbi.nlm.nih.gov, accessed on 12 July 2023) and ChemSpider (https://chemspider.com, accessed on 12 July 2023) databases, and structurally optimized and converted to PDB format using UCSF Chimera 1.17.1. The 3D structures of AKT1 (PDB ID 3o96) and VEGFA (PDB ID 6zfl) were downloaded from the RSCB PDB database (https://www.rcsb.org/, accessed on 12 July 2023), and water molecules and original ligands were removed using UCSF Chimera. AutoDockTools-1.5.6 was used to add polar hydrogen atoms and charges, the ligand rotation bond was established and the receptor and ligand were saved in PDBQT format. AutoDockVina was used to dock and search for the optimal conformation, which was determined by the binding mode and its corresponding binding affinity. Interactions between the compounds and targets were analyzed using LigPlot+ and Discovery Studio Visualizer 2021. For the redocking steps, the ligand was extracted and redocked into the original ligand binding pocket in the target protein, then the RMSD was calculated for each docking pose with respect to the native pose [17].

## 5. Conclusions

In conclusion, the result of this investigation increases the understanding of the molecular drivers that underlie AD progression and the prevention of the disease by marine polyphenols including EK, DK, and BK, and the identified key genes and pathways constitute potential therapeutic targets. Although more extensive experimental validation is required for the prevention and/or treatment of AD, these findings are expected to provide novel directions for the development of marine polyphenols and their potential use as a promising source of anti-AD agents.

## Figures and Tables

**Table 1:** Top 10 hub genes for marine polyphenols against Alzheimer’s disease.

Compound	Name	Degree	Average Shortest Path Length	Closeness Centrality	Clustering Coefficient
EK	AKT1	56	1.2821	0.7800	0.2130
	SRC	51	1.3462	0.7429	0.2965
	EGFR	49	1.3718	0.7290	0.2883
	MAPK3	48	1.3846	0.7222	0.2881
	ESR1	41	1.4744	0.6783	0.3610
	MAPK1	38	1.5128	0.6610	0.3826
	PIK3CA	30	1.6282	0.6142	0.4230
	HSP90AB1	29	1.6410	0.6094	0.3990
	MMP9	28	1.6410	0.6094	0.3677
	RAF1	26	1.6795	0.5954	0.5015
DK	VEGFA	18	1.8571	0.5385	0.3072
	PTGS2	17	1.8163	0.5506	0.2721
	HSP90AA1	17	1.9592	0.5104	0.3162
	JUN	16	1.9184	0.5213	0.3417
	PRKCB	11	2.0612	0.4851	0.3091
	PRKCD	11	2.0816	0.4804	0.5273
	PRKCA	11	1.9796	0.5052	0.4727
	SLC6A3	10	2.2245	0.4495	0.3111
	MAOB	10	2.4694	0.4050	0.5111
	HTR2C	9	2.2857	0.4375	0.2500
BK	AKT1	35	1.4068	0.7108	0.2487
	SRC	32	1.5254	0.6556	0.3367
	EGFR	31	1.5254	0.6556	0.3441
	ESR1	29	1.5593	0.6413	0.3744
	ERBB2	25	1.6780	0.5960	0.4500
	PTGS2	24	1.6271	0.6146	0.3877
	BCL2L1	21	1.7627	0.5673	0.4905
	PTK2	18	1.8305	0.5463	0.5294
	KDR	17	1.8136	0.5514	0.6324
	IGF1R	17	1.8475	0.5413	0.6838

**Table 2:** Molecular interaction between marine polyphenols and target proteins.

Group	Intersection Number	Intersection Elements
EK-BK	4	AKT1, SRC, EGFR, ESR1
DK-BK	1	PTGS2
EK	6	MAPK3, MAPK1, PIK3CA, HSP90AB1, MMP9, RAF1
DK	9	VEGFA, HSP90AA1, JUN, PRKCB, PRKCD, PRKCA, SLC6A3, MAOB, HTR2C
BK	5	ERBB2, BCL2L1, PTK2, KDR, IGF1R

**Table 3:** The common pathways of marine polyphenols.

Term	Pathway	*p*-Value
Hsa05200	Pathways in cancer	3.04 × 10^−6^
Hsa01521	EGFR tyrosine kinase inhibitor resistance	1.26 × 10^−4^
Hsa04010	MAPK signaling pathway	9.23 × 10^−5^
Hsa04015	Rap1 signaling pathway	4.26 × 10^−5^
Hsa04014	Ras signaling pathway	1.33 × 10^−5^
Hsa05207	Chemical carcinogenesis—receptor activation	4.56 × 10^−5^
Hsa04510	Focal adhesion	3.34 × 10^−5^

**Table 4:** Molecular docking of marine polyphenols with key targets.

Compound- Target Gene	Binding Affinity (kcal/mol)	No. of Pi-Interaction	No. of H-Bond	H-Bonding Residues	Length of H-Bond (Å)	van der Waals Residues	
EK-AKT1	−9.7	6	7	Gln79, Thr81, Thr82, Ser205, Thr211, Thr291	3.15, 3.10/3.0 2.79, 3.01, 3.06, 3.18	Trp80, Leu210, Leu264, Lys268, Val270, Tyr272, Asp292	
DK-VEGFA	−10.1	5	6	Glu30, Ser50, Gly59, Asn62, Asp63, Lys107	2.86, 3.04, 2.74, 2.98, 2.92/3.10 3.14	Ile29, Glu30, Thr31, Leu32, Cys57, Gly58, Cys60, Cys61, Glu64, Leu66, Glu67, Cys68,	
BK-AKT1	−11.3	5	5	Gln79, Asn199, Arg273, Thr291, Cys296	2.60, 2.83, 2.86, 3.15, 2.72	Asn53, Asn54, Ala58, Gln59, Trp80, Thr82, Val201, Val270, Tyr272, Asp274, Asp292, Gly294	

## Data Availability

The data are provided in the manuscript.

## Supplementary Materials

The following supporting information can be downloaded at: https://www.mdpi.com/article/10.3390/md21110580/s1, Table S1: The list of potential targets for eckol(EK), dieckol(DK), and 8,8′-bieckol(BK); Table S2: The list of Alzheimer’s disease related targets from GeneCards; Table S3: The list of biological processes (BP) category of EK from GO enrichment analysis; Table S4: The list of cellular components (CC) category of EK from GO enrichment analysis; Table S5: The list of molecular function (MF) categories of EK from GO enrichment analysis; Table S6: The list of BP categories of DK from GO enrichment analysis; Table S7: The list of CC categories of DK from GO enrichment analysis; Table S8: The list of MF categories of DK from GO enrichment analysis; Table S9: The list of BP categories of BK from GO enrichment analysis; Table S10: The list of CC categories of BK from GO enrichment analysis; Table S11: The list of MF categories of BK from GO enrichment analysis; Table S12: The list of top 20 pathways of marine polyphenols from KEGG pathway analysis.
